# *Mycobacterium ulcerans* DNA Not Detected in Faecal Samples from Buruli Ulcer Patients: Results of a Pilot Study

**DOI:** 10.1371/journal.pone.0019611

**Published:** 2011-05-04

**Authors:** Fred S. Sarfo, Caroline J. Lavender, Janet A. M. Fyfe, Paul D. R. Johnson, Timothy P. Stinear, Richard O. Phillips

**Affiliations:** 1 Komfo Anokye Teaching Hospital, Kumasi, Ghana; 2 Mycobacterium Reference Laboratory, Victorian Infectious Diseases Reference Laboratory, North Melbourne, Victoria, Australia; 3 Department of Infectious Diseases, Austin Health, Heidelberg, Victoria, Australia; 4 Department of Microbiology and Immunology, The University of Melbourne, Parkville, Victoria, Australia; 5 Department of Microbiology, Monash University, Clayton, Victoria, Australia; Fundació Institut Germans Trias i Pujol; Universitat Autònoma de Barcelona CibeRES, Spain

## Abstract

It has recently been shown that in a Buruli ulcer (BU) endemic region of southeastern Australia, significant numbers of possums (native tree-dwelling marsupials) have clinical BU disease. Furthermore, based on quantitative PCR (qPCR) analysis, animals with BU lesions (and some without) shed *M. ulcerans* DNA in their faeces, indicative of bacterial loads of up to 10^8^ organisms/gram. These findings led us to propose that humans might also harbour *M. ulcerans* in their gastrointestinal tract and shed the bacterium in their faeces. We conducted a pilot study and collected faecal swabs from 26 patients with confirmed BU and 31 healthy household controls. Faecal samples were also collected from 10 healthy controls from non-endemic regions in Ghana. All 67 specimens were negative when tested by IS*2404* PCR. The detection sensitivity of this method was ≥10^4^ bacteria per gram (wet-weight) of human faecal material. We conclude that the human gastrointestinal tract is unlikely to be a significant reservoir of *M. ulcerans*.

## Introduction

Efforts to control the spread of Buruli ulcer (BU) will be significantly improved if we understand the ecology of the causative agent, *Mycobacterium ulcerans*. Recent surveys of the environment in BU endemic regions near Melbourne, Australia, have revealed that over 40% of possums (small tree-dwelling animals native to Australia with a body temperature like humans of ∼36°C) are shedding high levels of *M. ulcerans* DNA in their faeces and may be acting as reservoirs of *M. ulcerans* [1]. A transmission model is proposed where contaminated possum excreta enters mosquito breeding habitats (drains and roof gutters near houses) where the insects may acquire the bacterium and then transmit it to humans during biting [2], [3]. There are no possums in Africa and a recent survey of small animals in Benin did not identify any species with *M. ulcerans* in their organs or faeces [4]. Nevertheless, the presence of *M. ulcerans* in the gastrointestinal tract of warm-blooded, terrestrial animals has led to a reconsideration of where *M. ulcerans* is located in the environment, and raises the possibility of a terrestrial animal reservoir for the bacterium in African BU endemic regions. We therefore speculated that humans were acting like the possums - as both a disease-susceptible host and possible reservoir. To test this hypothesis we used IS*2404* qPCR to screen faecal specimens from confirmed BU patients, household contacts, and control samples from BU non-endemic regions in Ghana for the presence of *M. ulcerans* DNA.

## Methods

### Clinical Setting

Tepa Government Hospital, Ahafo North District and Komfo Anokye Teaching Hospital Hospital, Kumasi.

### Clinical specimen collection

In this pilot study, we assembled a convenience sample of patients and accompanying unaffected household members who were attending a Buruli treatment clinic. Patients and controls were recruited by local health workers in villages near Tepa Government Hospital in the Ashanti region of Ghana where there is a high prevalence of *M. ulcerans* disease. All patients enrolled in this study met the WHO case definition for BU. Non-endemic control subjects were recruited from villages where BU has never been reported. Relevant details for patients and controls are summarized in Tables 1, 2, 3. Fine needle aspirates and swab specimens were taken from patients to confirm the clinical diagnosis by IS*2404* PCR. Patients were then treated with 10 mg/kg oral rifampicin and 15 mg/kg intramuscular streptomycin combination daily for 8 weeks (SR8), administered at village health posts. Faecal samples were collected in sterile 50 ml BD plaster containers and transported cold to the laboratory at the Komfo Anokye Teaching Hospital and stored at 4°C. For four BU patients, faecal samples were obtained during antibiotic treatment. Swabs of faecal samples were subsequently shipped to the WHO Collaborating Centre for *Mycobacterium ulcerans* in Australia for detection and quantification of *M. ulcerans* by qPCR.

**Table 1:** Clinical details for BU patients enrolled in the study.

Patient Reference No	Antibiotic Treatment	Sex	Age	Endemic District	Disease Form/Category#	Laboratory Confirmation(PCR, Culture, ZN)
M101	6 weeks, STR*	M	10	Asutifi	E/IIII	IS2404 PCR +
M107	2 weeks, STR	M	11	Ahafo Ano North	E/III	IS2404 PCR +
M101	6 weeks, STR	M	10	Asutifi	E/III	IS2404 PCR +
M102	4 weeks, STR	M	32	Ahafo Ano North	N/I	IS2404 PCR +
M98	None	M	60	Asutifi	U/II	IS2404 PCR +, Culture +
M104	None	M	13	Asutifi	U/II	IS2404 PCR +
M105	None	M	11	Ahafo Ano North	E/III	IS2404 PCR +
M107	None	F	13	Ahafo Ano North	U/III	IS2404 PCR +
M110	None	M	2	Ahafo Ano North	N/I	IS2404 PCR +
M111	None	M	29	Asutifi	Q/II	IS2404 PCR +
M112	None	F	20	Asutifi	U/II	IS2404 PCR +
M113	None	F	3	Ahafo Ano North	E/II	IS2404 PCR +
M116	None	F	37	Ahafo Ano North	N/I	IS2404 PCR +
M119	None	M	14	Ahafo Ano North	N/I	IS2404 PCR +
M121	None	M	16	Ahafo Ano North	U/II	IS2404 PCR +
M123	None	F	9	Ahafo Ano North	U/I	IS2404 PCR +
M124	None	F	Un-known	Tano North	U/III	IS2404 PCR +, Culture +, ZN+
M126	None	M	14	Ahafo Ano North	U/III	IS2404 PCR +
M127	None	F	32	Tano North	U/III	IS2404 PCR +, Culture +, ZN +
M128	None	M	3	Asutifi	U/III	IS2404 PCR +
M129	None	M	18	Asutifi	E/III	IS2404 PCR +
M131	None	M	8	Asutifi	Q/II	IS2404 PCR +
M132	None	M	11	Asutifi	E/III	IS2404 PCR +
M133	None	M	34	Ahafo Ano North	U/III	IS2404 PCR +
M84	None	F	10	Ahafo Ano North	U/II	IS2404 PCR +
M96	None	F	1	Tano North	U/III	IS2404 PCR +

*STR = WHO recommended treatment of 8 weeks streptomycin and rifampicin;

# ‘Category’ refers to WHO clinical severity scoring system for BU E = edematous, N = nodule, Q = Plaque, U = ulcerative.

**Table 2:** Details for household contacts enrolled in the study.

Ref. No.	Sex	Age	Endemic District
9	M	3	Asutifi
18	M	8	Asutifi
19	F	Unknown	Tano North
29	M	8	Asutifi
41	M	18	Asutifi
42	F	13	Asutifi
44	F	16	Ahafo Ano North
45	F	23	Asutifi
50	M	14	Asutifi
51	M	11	Asutifi
53	F	13	Ahafo Ano North
56	F	32	Tano North
64	M	14	Ahafo Ano North
66	M	16	Ahafo Ano North
68	F	13	Ahafo Ano North
57	M	17	Ahafo Ano North
32	F	40	Ahafo Ano North
43	F	14	Ahafo Ano North
34	M	16	Tano North
47	F	36	Ahafo Ano North
36	F	82	Tano North
59	F	14	Asutifi
35	M	45	Asutifi
67	F	15	Asutifi
69	M	32	Asutifi
61	F	19	Ahafo Ano North
39	F	23	Ahafo Ano North
40	F	82	Tano North
33	F	5	Asutifi
37	F	24	Ahafo Ano North
38	M	46	Ahafo Ano North

**Table 3:** Details for non-exposed controls enrolled in the study.

Ref. No.	Sex	Age	District
82	F	16	Asafo
83	M	14	Asuoyeboa
84	M	26	Brofoyedu
85	F	29	Anyinam
86	M	15	Asouase
87	M	17	Suame
88	M	28	New Tafo
89	F	30	Santasi
90	M	13	Asafo
91	M	8	Bantama

### Faecal spiking experiment

In an experiment to mimic testing swabs of human faecal material, five equal aliquots of human faeces (partially liquefied by the addition of water to aid homogenization) were spiked with a 10-fold dilution series of *M. ulcerans* equivalent to 3.2×10^5^ organisms per gram, and a sixth aliquot was included as an unspiked control. Bacterial numbers were estimated based on the turbidity of the initial suspension, which was equivalent to a MacFarlane Standard of 1. Sterile swabs were used to transfer spiked faecal material to glass bead bottles containing 2 ml of phosphate buffered saline (PBS). Weighing tubes containing the spiked faecal samples before and after swabbing indicated an average 40 mg (range: 30–60 mg) of faecal material was transferred by this technique. *M ulcerans* DNA was extracted as described below.

### Quantitative PCR analysis

DNA was extracted from faecal samples using the FastDNA® SPIN Kit for Soil and FastPrep® Instrument (Qbiogene Inc., Carlsbad, CA) in combination with the QIAxtractor Instrument (QIAGEN Pty Ltd, Doncaster, Victoria, Australia). Swab ends were placed in sterile bead bottles with 2 ml of phosphate buffered saline (PBS) and vortexed vigorously for 2 minutes. One millilitre of sample was transferred to Lysing Matrix E tubes and centrifuged at 16,000× *g* for 10 mins. The supernatant was removed and samples were processed according to the FastDNA® SPIN Kit for Soil protocol with the following modification. After the Protein Precipitating Solution (PPS) was added to the lysate and the samples were centrifuged, 200 µl of sample was added to the QIAxtractor Instrument and purified using the QIAxtractor Liquid Sample Protocol. To exclude the possibility of cross-contamination, at least one negative control was included in every DNA extraction. DNA extracts were tested for the presence of *M. ulcerans* DNA using a TaqMan real-time PCR assay targeting IS*2404* as described previously [5]. The assay is multiplexed with an internal positive control (IPC) to monitor PCR inhibition and control for false negative results. Two positive controls and four negative (no template) controls were included in every real-time PCR assay to ensure the test results were valid. Laboratory technicians were not aware of the origin of the samples they were testing.

### Ethics statement

The Committee on Human Research, Publication and Ethics (CHRPE) at the School of Medical Sciences, Kwame Nkrumah University of Science and Technology, Kumasi, Ghana approved the study protocol. Informed, written consent was obtained from all participants or their legal guardians as approved by the CHRPE.

## Results

**Figure 1:**
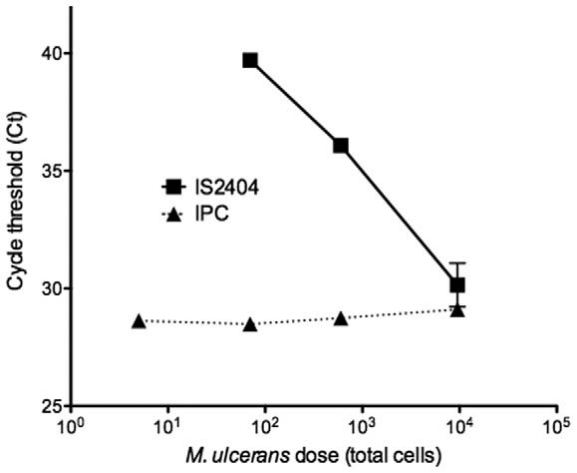
Results of spiking experiment, showing sensitivity of IS*2404* qPCR for the detection of *M. ulcerans* in human faecal material. Depicted is the mean and standard deviation of duplicate samples. IPC: Internal Positive Control.

Prior to PCR screening of human faecal material we established the detection sensitivity of DNA extraction and IS*2404* qPCR analysis method. Spiking experiments with serial 10-fold dilutions of *M. ulcerans* in human faeces showed that the smallest inoculum of *M. ulcerans* that was readily detected by IS*2404* qPCR was 1.2×10^3^ bacterial cells per swab (Fig. 1), which represents an overall minimum detection limit ≥3×10^4^ *M. ulcerans* per gram wet faecal material. PCR inhibitors were effectively removed by this DNA extraction method as the IPC was readily detected in DNA extracted from all dilutions of spiked faecal material (Figure 1).

**Figure 2:**
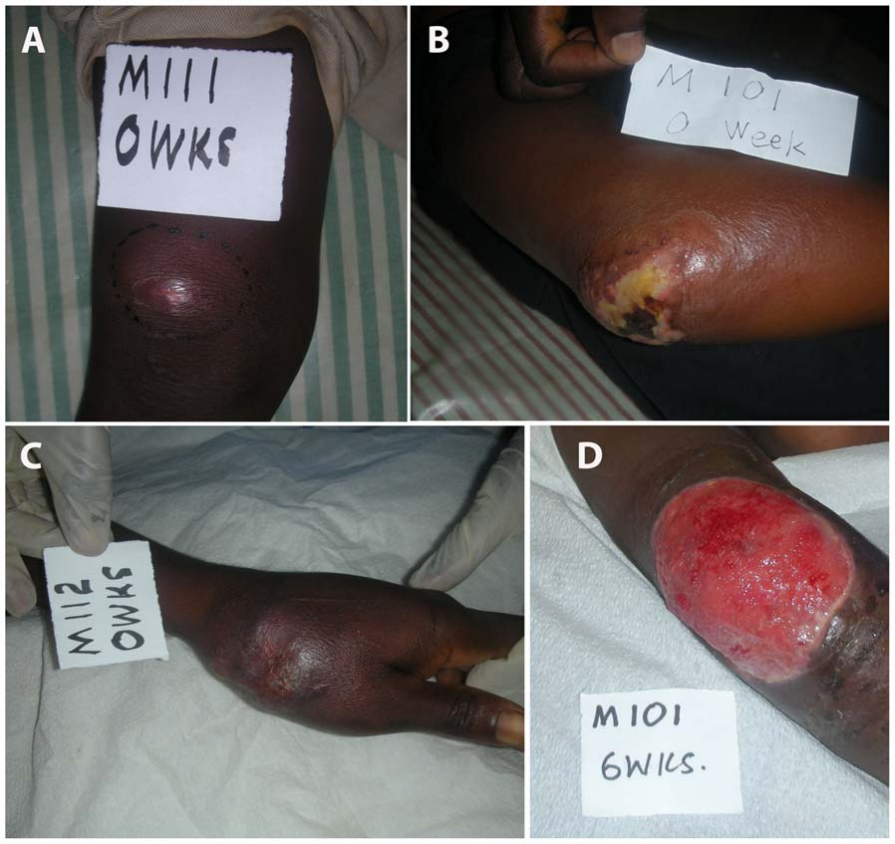
Clinical presentation of three, confirmed BU patients enrolled in this study showing: (A) nodule of the right knee; (B) ulcer of the right elbow; (C) ulcer of the left hand at diagnosis; and (D) ulcer of the right elbow 6 weeks post diagnosis and commencement of SR8 with surgical intervention.

Having established a robust molecular detection assay, faecal swabs were collected from 26 PCR-confirmed BU patients (age range 2–37 years, 17 male subjects & 9 female subjects) and 31 healthy household contacts (age range 3–82 years, 13 male subjects & 18 female subjects) (Tables 1, 2). The patients and contacts came from two BU endemic regions in Ghana (Ashanti and Brong Ahafo districts). Faecal swabs were also collected from 10 healthy controls from nine non-endemic regions in Ghana (age range 8–30 years, 7 male subjects & 3 female subjects) (Table 3). Four of the 26 BU patients had commenced SR8 at the time of sample collection. None of the other study participants were on any antimicrobial treatment. Clinical presentation among the patient cohort encompassed the spectrum of BU with the exception of bone involvement (Table 1, Figure 2). Total DNA was extracted from all 67 specimens and tested by IS*2404* qPCR. All 67 samples were IS*2404* PCR negative. Based on our assessments of assay sensitivity, this indicates there were <10^4^ *M. ulcerans* per gram faecal material. The results are not explained by PCR inhibition as the internal positive control was amplified from each of the 67 samples at a cycle threshold value equivalent to that obtained in both positive and negative controls.

## Discussion

The data collected in this pilot study suggest that, unlike possums in a BU endemic area (which may shed up to 10^8^ organisms per gram of faeces), humans do not shed detectable levels of *M. ulcerans* in their faeces. Nevertheless, even though humans do not appear to carry *M. ulcerans* in their gastrointestinal tracts, they may act as a susceptible host and a reservoir of *M. ulcerans*. With the exception of possums in Australia, human BU lesions represent the highest concentration of *M. ulcerans* cells in any source identified so far. Bacteria from human BU lesions may be shed into the environment by various means, such as household fomite contamination, shedding into waterways during bathing of lesions and/or disseminated by insects. High resolution molecular epidemiological analysis of strains from the Densu River Basin in Ghana suggest that transmission of *M. ulcerans* occurs at a very local level, consistent with a local reservoir of the bacterium [6]. Furthermore, a recent study in Benin of the seroepidemiological potential of *M. ulcerans* antigens suggested that there is widespread exposure to *M. ulcerans* in BU endemic areas, again consistent with a significant local reservoir of the bacterium in close contact with humans [7].

This investigation suggests that humans are unlikely to carry *M. ulcerans* in significant quantities in their gastrointestinal tracts. However, it is also important to recognize that this is a pilot study with only 26 BU patients enrolled by convenience from two endemic foci in Ghana. Studies of a larger and randomly selected patient/control cohort, within and between different BU endemic countries, and across all manifestations of BU are now warranted. While it is possible that human BU lesions themselves could be a major indirect source of new human infections, the search should also continue for another animal source of *M. ulcerans* in Africa like possums in southern Australia.
